# Supplementary material for: Drug mechanism‐of‐action discovery through the integration of pharmacological and CRISPR screens
Source: Mol Syst Biol. 2020 Jul 6;16(7):e9405. doi: 10.15252/msb.20199405 (PMC7336273; doi:10.15252/msb.20199405)
Supplement: Supplementary file 1 — Expanded View Figures PDF [file MSB-16-e9405-s001.pdf]

Expanded View Figures

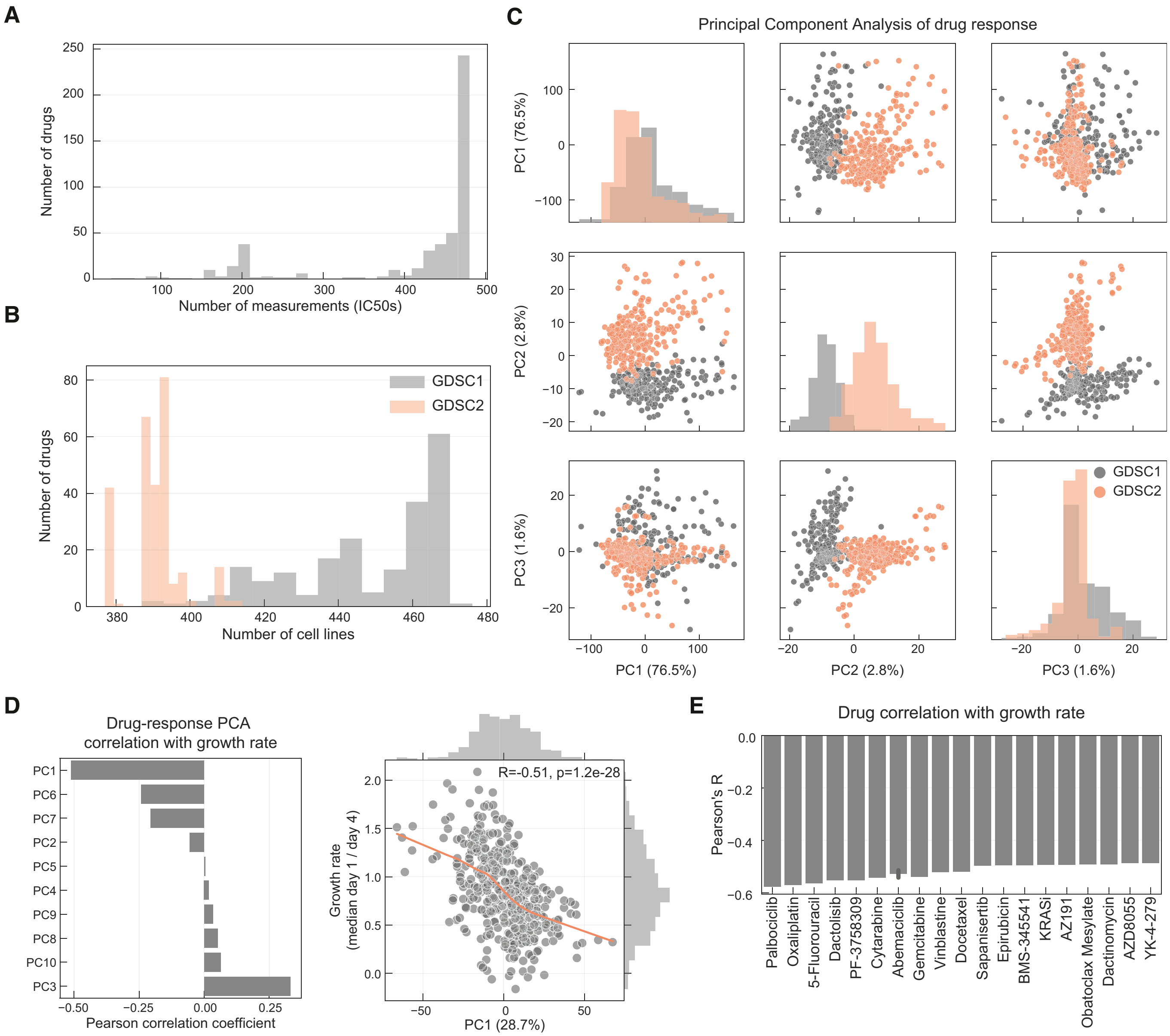

Figure EV1. Overview of the drug sensitivity data sets.

A Histogram of the number of IC_{50} values measured per drug.
B Number of drugs measured per cell line in each pharmacological data set.
C PCA analysis of the drug response measurements separated by GDSC1 and GDSC2.
D Pearson correlation coefficient between each principal component (PC) and cell lines growth rate.
E Top absolutely correlated drugs with growth rate.

© 2020 The Authors

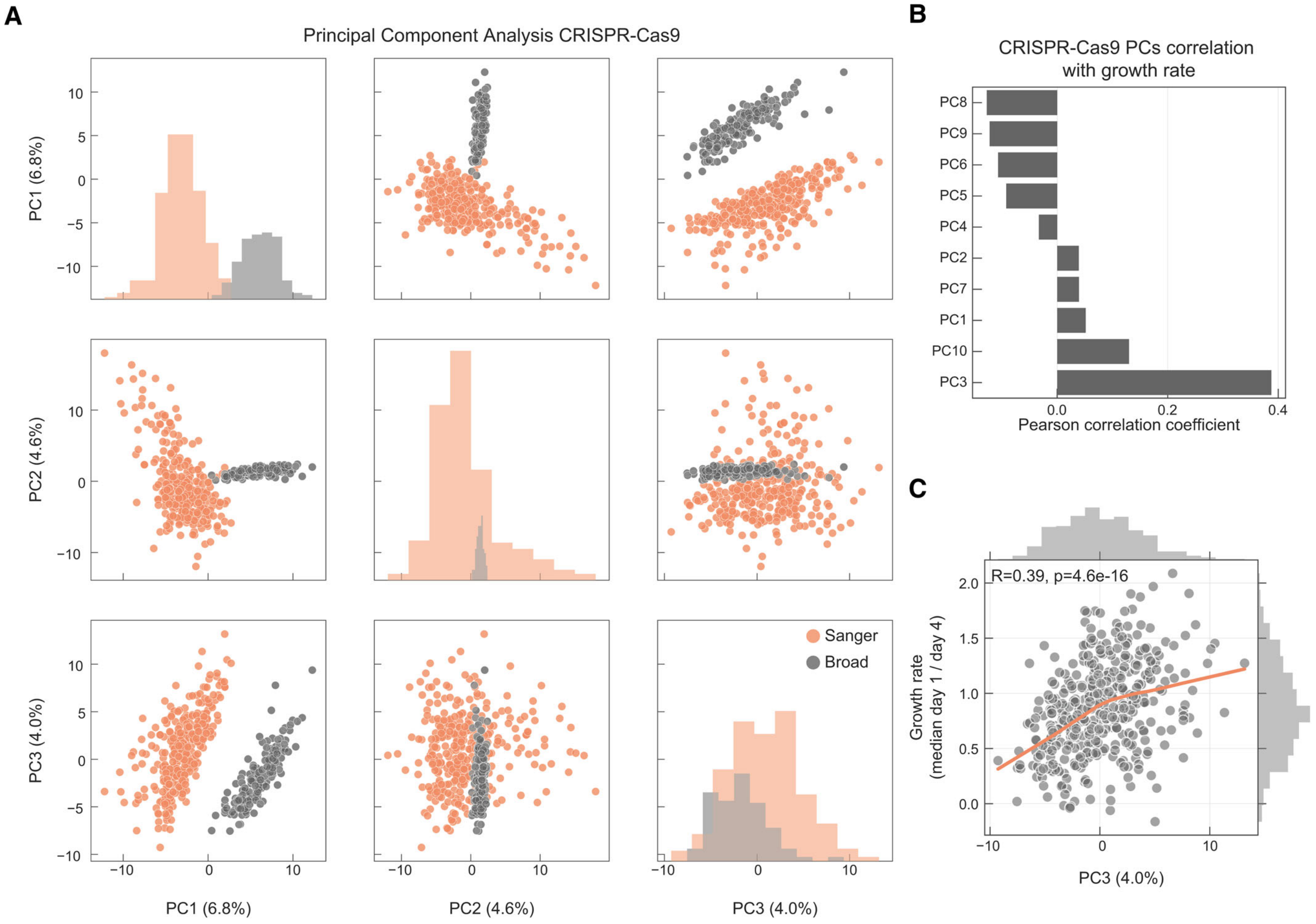

Figure EV2. Overview of the CRISPR-Cas9 data sets.

A PCA analysis of the samples in the CRISPR-Cas9 screens, samples institute of origin is highlighted.
B Correlation coefficients between all top 10 PCs and growth rate.
C Correlation between cell lines growth rate and PC3 (Pearson correlation coefficient reported in the top left).

 © 2020 The Authors

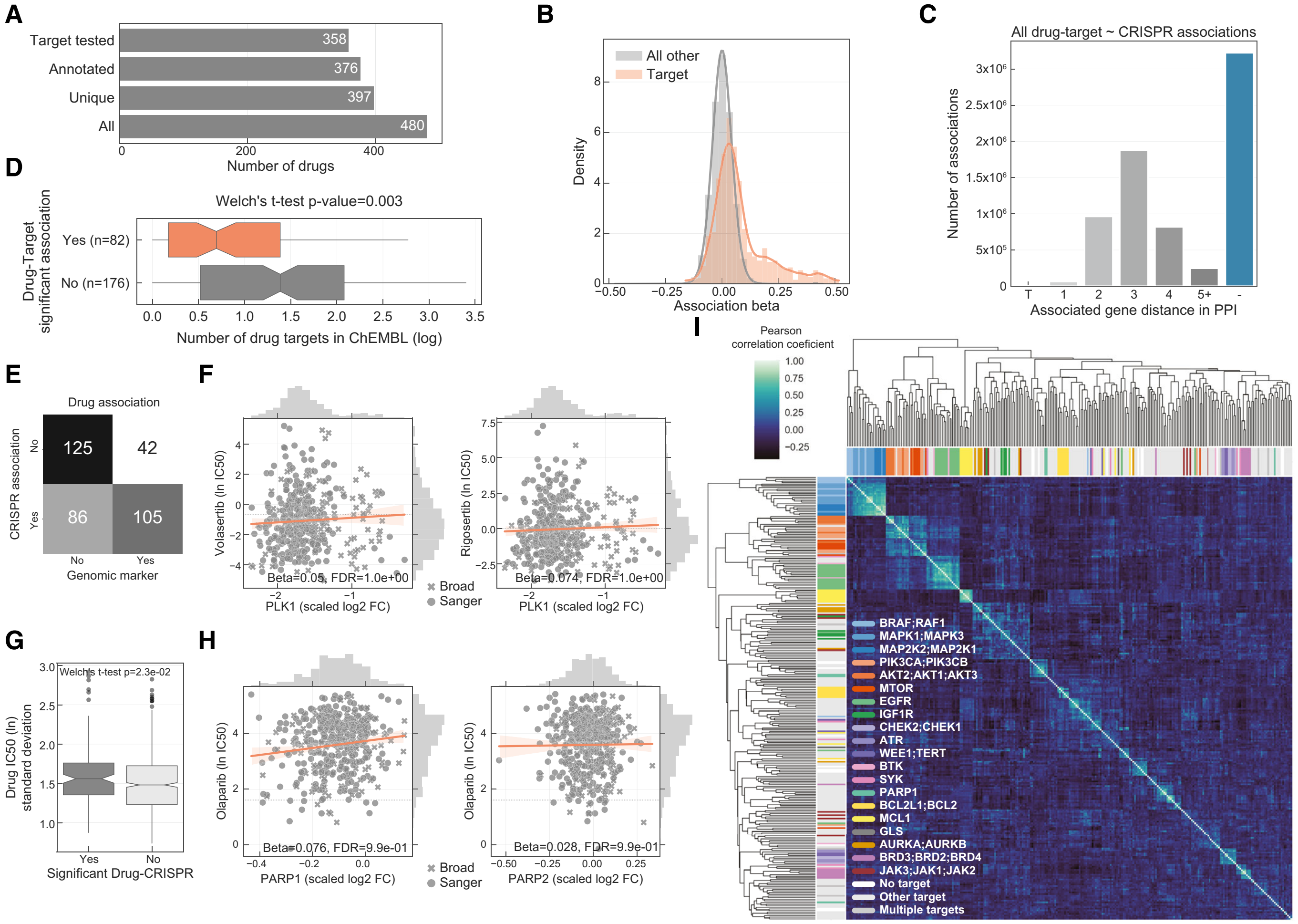

Figure EV3. Drug response and gene fitness associations.

A Total number of drugs utilised in the study and the different levels of information available: "All" represents all the drugs including replicates screened with different technologies (GDSC1 and GDSC2); "Unique" counts the number of unique drug names; "Annotated" shows the number of unique drugs with manual annotation of nominal targets; and "Target tested" represents the number of unique drugs, with target information, for which the target has been knocked-out in the CRISPR-Cas9 screens.

B Histogram of the drug–gene associations effect sizes (beta) highlighting drug–target associations.

C Distribution of the shortest path lengths between all the tested drug–gene pairs. For drugs with multiple targets the smallest shortest path of all the targets was taken. T represents the drug target and "—" represents no link was found.

D Distribution of number of drug targets found in an unsupervised way using the ChEMBL database. Drugs are grouped by having significant drug–target associations. Box-and-whisker plots show 1.5× interquartile ranges and 5–95th percentiles, centres indicate medians.

E Contingency matrix of significant drug associations with CRISPR fold changes and binarised event matrix of genomic features, i.e. mutations and copy number gain or loss.

F PLK1 inhibitors drug response correlation with PLK1 knockout log_2 fold change (FC) gene fitness effects. The dashed grey line indicates the dose response highest drug concentration.

G Drug–target associations split by significance (FDR-adjusted likelihood-ratio test *P*-value < 10%) plotted against the standard deviation of the drug IC_{50} (ln) measurements of the respective pair (significant "Yes" n = 129, significant "No" n = 684). Box-and-whisker plots show 1.5× interquartile ranges and 5–95th percentiles, centres indicate medians.

H Similar to (F), correlation of olaparib drug response and both targets PARP1 and PARP2 gene fitness effects.

I Correlation heatmap of the drug–gene effect size across all the genes. Drugs are coloured according to their targets.

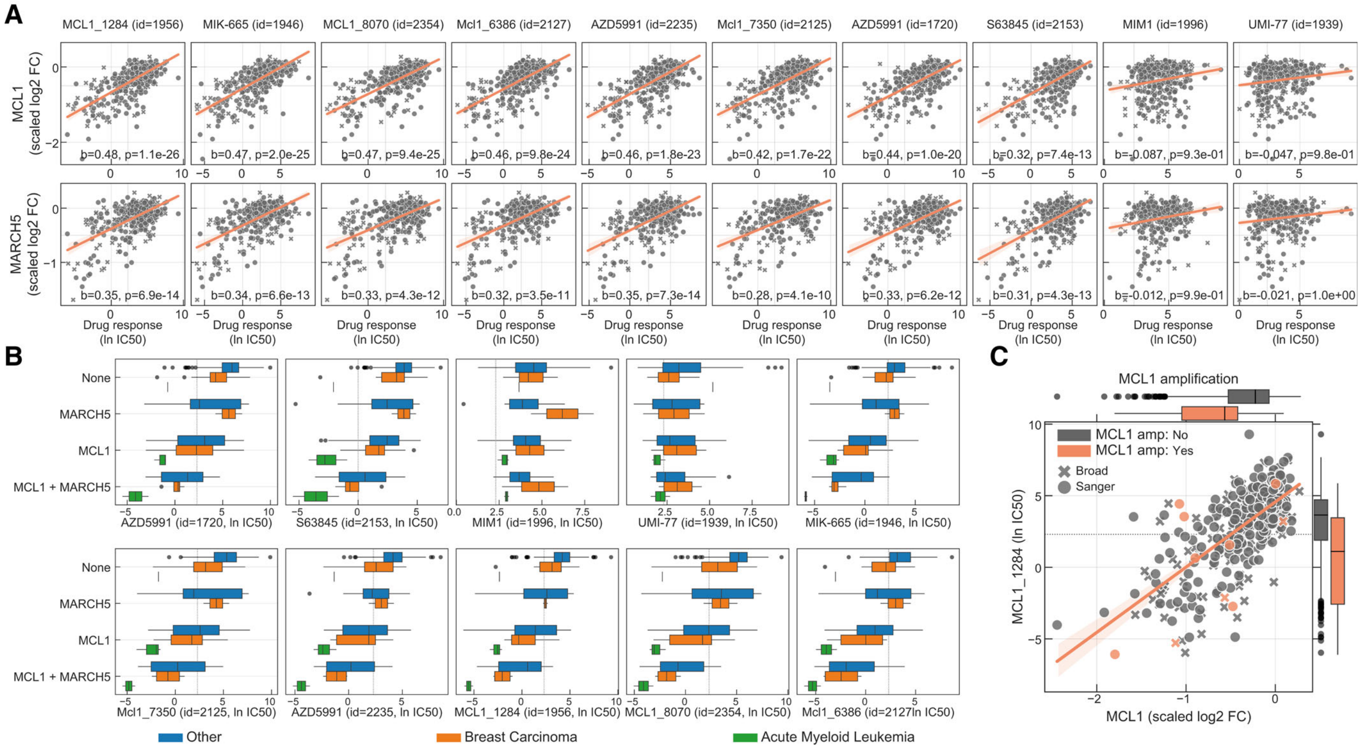

Figure EV4. MCL1 inhibitors.

A Correlation of all MCL1 inhibitor IC_{50} values against MCL1 and MARCH5 gene fitness profiles. Effect sizes (*b*) and FDR (*P*) of the association are reported on the bottom.

B Stratification of the MCL1 inhibitors drug response measurements according to the cell line dependency on MARCH5 and/or MCL1. Gene vulnerabilities are independent from each other, meaning knockouts were introduced independently and not at the same time. Responses are then split according to the cancer type of the cell lines. Number of measurements per boxplot are: acute myeloid leukaemia (MCL1 = 3, MCL1 + MARCH5 = 2, None = 1), breast carcinoma (MARCH5 = 2, MCL1 = 9, MCL1 + MARCH5 = 4, None = 14) and Other (MARCH5 = 9, MCL1 = 52, MCL1 + MARCH5 = 21, None = 236). Vulnerable cell lines to MARCH5 and MCL1 knockout were defined as those with a depletion of at least 50% of that visible for essential genes on the particular cell line (scaled $\log_2$ fold change < -0.5). Grey dashed line represents the maximum concentration used in the dosage response curve of the respective drug. Box-and-whisker plots show 1.5× interquartile ranges and 5–95th percentiles, centres indicate medians.

C Representative example of a MCL1 inhibitor and their relation with MCL1 gene fitness, with cell lines containing copy number amplification of MCL1 highlighted in orange. Copy number amplified cells were defined taking into consideration their ploidy status, cells with (ploidy $\leq$ 2.7 and copy number $\geq$ 5) or (ploidy $>$ 2.7 and copy number $\geq$ 9) were considered as having MCL1 amplified. Box-and-whisker plots show 1.5× interquartile ranges and 5–95th percentiles, centres indicate medians.

Figure EV5. Robust pharmacological associations.

A Most frequent genomic alterations across the cancer cell lines.

B, C Most significant associations between (B), genomic features and (C), gene expression profiles with drug response and gene fitness.

D Number of significant drug–gene pairs across the different types of interactions. Drug–gene pairs were categorised considering the shortest path length between the drug target(s) and the associated gene. T represents the drug target and "—" represents no link was found.

E Robust pharmacological association between the expression of BCL2L1 and the significantly correlated pair of MCL1_1284 drug and MCL1 gene fitness profile.

F Similarly to (E), but instead it represents a robust pharmacological association between BAX and MDM2 and Nutlin-3a.

▶

 © 2020 The Authors

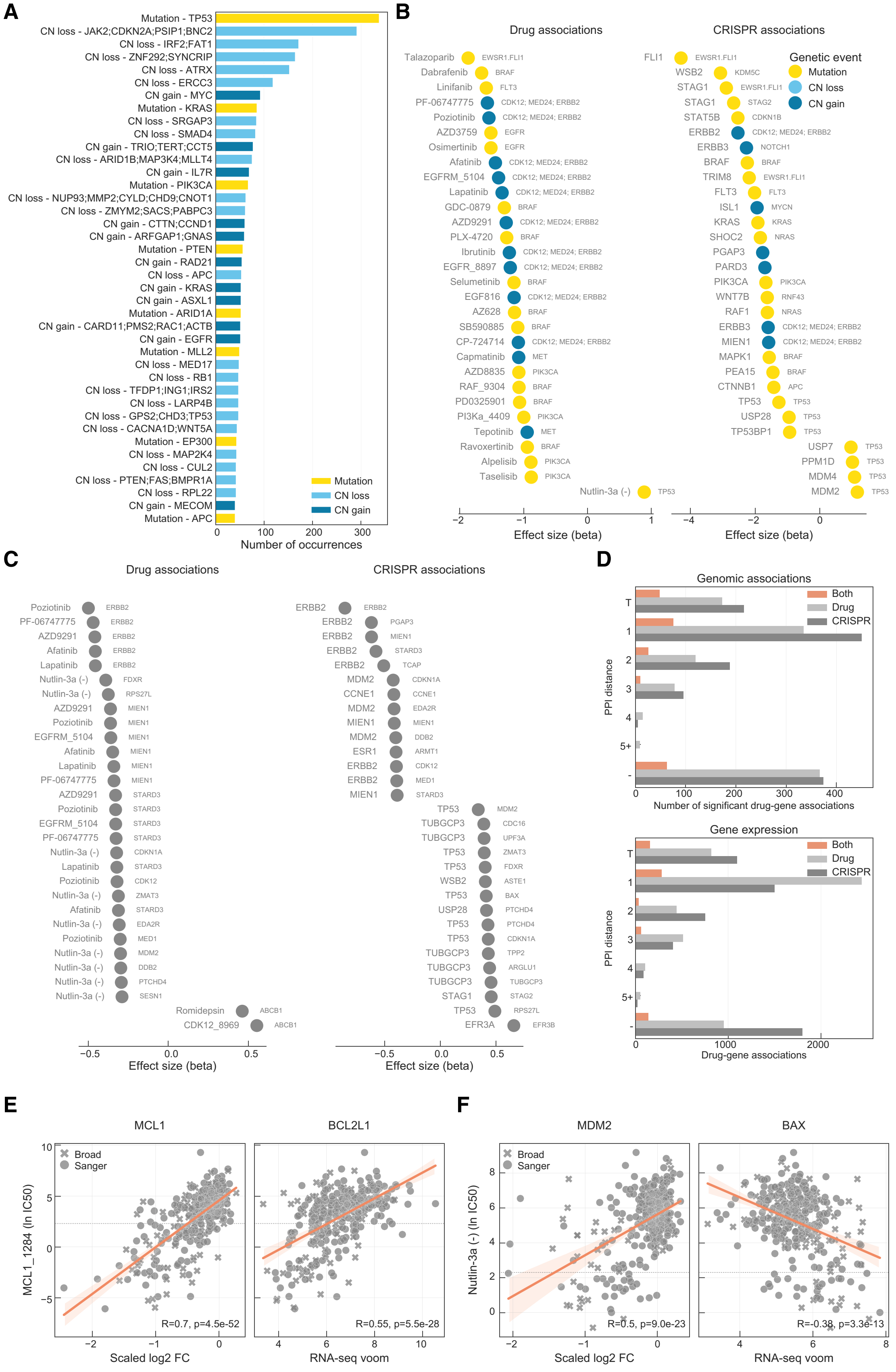
A
Mutation - TP53
CN loss - JAK2;CDKN2A;PSIP1;BNC2
CN loss - IRF2;FAT1
CN loss - ZNF292;SYNCRIP
CN loss - ATRX
CN loss - ERCC3
CN gain - MYC
Mutation - KRAS
CN loss - SRGAP3
CN loss - SMAD4
CN gain - TRIO;TERT;CCT5
CN loss - ARID1B;MAP3K4;MLLT4
CN gain - IL7R
Mutation - PIK3CA
CN loss - NUP93;MMP2;CYLD;CHD9;CNOT1
CN loss - ZMYM2;SACS;PABPC3
CN gain - CTTN;CCND1
CN gain - ARFGAP1;GNAS
Mutation - PTEN
CN gain - RAD21
CN loss - APC
CN gain - KRAS
CN gain - ASXL1
Mutation - ARID1A
CN gain - CARD11;PMS2;RAC1;ACTB
CN gain - EGFR
Mutation - MLL2
CN loss - MED17
CN loss - RB1
CN loss - TFDP1;ING1;IRS2
CN loss - LARP4B
CN loss - GPS2;CHD3;TP53
CN loss - CACNA1D;WNT5A
Mutation - EP300
CN loss - MAP2K4
CN loss - CUL2
CN loss - PTEN;FAS;BMPR1A
CN loss - RPL22
CN gain - MECOM
Mutation - APC
Mutation
CN loss
CN gain
0 100 200 300
Number of occurrences
B
Drug associations
Talazoparib EWSR1.FLI1
Dabrafenib BRAF
Linifanib FLT3
PF-06747775 CDK12; MED24; ERBB2
Poziotinib CDK12; MED24; ERBB2
AZD3759 EGFR
Osimertinib EGFR
Afatinib CDK12; MED24; ERBB2
EGFRM_5104 CDK12; MED24; ERBB2
Lapatinib CDK12; MED24; ERBB2
GDC-0879 BRAF
AZD9291 CDK12; MED24; ERBB2
PLX-4720 BRAF
Ibrutinib CDK12; MED24; ERBB2
EGFR_8897 CDK12; MED24; ERBB2
Selumetinib BRAF
EGF816 CDK12; MED24; ERBB2
AZ628 BRAF
SB590885 BRAF
CP-724714 CDK12; MED24; ERBB2
Capmatinib MET
AZD8835 PIK3CA
RAF_9304 BRAF
PD0325901 BRAF
PI3Ka_4409 PIK3CA
Tepotinib MET
Ravoxertinib BRAF
Alpelisib PIK3CA
Taselisib PIK3CA
Nutlin-3a (-) TP53
−2 −1 0 1
Effect size (beta)
CRISPR associations
FLI1 EWSR1.FLI1
WSB2 KDM5C
STAG1 EWSR1.FLI1
STAG1 STAG2
STAT5B CDKN1B
ERBB2 CDK12; MED24; ERBB2
ERBB3 NOTCH1
BRAF BRAF
TRIM8 EWSR1.FLI1
FLT3 FLT3
ISL1 MYCN
KRAS KRAS
SHOC2 NRAS
PGAP3
PARD3
PIK3CA PIK3CA
WNT7B RNF43
RAF1 NRAS
ERBB3 CDK12; MED24; ERBB2
MIEN1 CDK12; MED24; ERBB2
MAPK1 BRAF
PEA15 BRAF
CTNNB1 APC
TP53 TP53
USP28 TP53
TP53BP1 TP53
USP7 TP53
PPM1D TP53
MDM4 TP53
MDM2 TP53
Genetic event
Mutation
CN loss
CN gain
−4 −2 0
Effect size (beta)
C
Drug associations
Poziotinib ERBB2
PF-06747775 ERBB2
AZD9291 ERBB2
Afatinib ERBB2
Lapatinib ERBB2
Nutlin-3a (-) FDXR
Nutlin-3a (-) RPS27L
AZD9291 MIEN1
Poziotinib MIEN1
EGFRM_5104 MIEN1
Afatinib MIEN1
Lapatinib MIEN1
PF-06747775 MIEN1
AZD9291 STARD3
Poziotinib STARD3
EGFRM_5104 STARD3
PF-06747775 STARD3
Nutlin-3a (-) CDKN1A
Lapatinib STARD3
Poziotinib CDK12
Nutlin-3a (-) ZMAT3
Afatinib STARD3
Nutlin-3a (-) EDA2R
Poziotinib MED1
Nutlin-3a (-) MDM2
Nutlin-3a (-) DDB2
Nutlin-3a (-) PTCHD4
Nutlin-3a (-) SESN1
Romidepsin ABCB1
CDK12_8969 ABCB1
−0.5 0.0 0.5
Effect size (beta)
CRISPR associations
ERBB2 ERBB2
ERBB2 PGAP3
ERBB2 MIEN1
ERBB2 STARD3
ERBB2 TCAP
MDM2 CDKN1A
CCNE1 CCNE1
MDM2 EDA2R
MIEN1 MIEN1
MDM2 DDB2
ESR1 ARMT1
ERBB2 CDK12
ERBB2 MED1
MIEN1 STARD3
TP53 MDM2
TUBGCP3 CDC16
TUBGCP3 UPF3A
TP53 ZMAT3
TP53 FDXR
WSB2 ASTE1
TP53 BAX
USP28 PTCHD4
TP53 PTCHD4
TP53 CDKN1A
TUBGCP3 TPP2
TUBGCP3 ARGLU1
TUBGCP3 TUBGCP3
STAG1 STAG2
TP53 RPS27L
EFR3A EFR3B
−0.5 0.0 0.5
Effect size (beta)
D
Genomic associations
Both
Drug
CRISPR
T
1
2
3
4
5+
-
PPI distance
0 100 200 300 400
Number of significant drug-gene associations
Gene expression
Both
Drug
CRISPR
T
1
2
3
4
5+
-
PPI distance
0 1000 2000
Drug-gene associations
E
MCL1
Broad
Sanger
MCL1_1284 (ln IC50)
10 5 0 −5
R=0.7, p=4.5e-52
−2 −1 0
Scaled log2 FC
BCL2L1
R=0.55, p=5.5e-28
4 6 8 10
RNA-seq voom
F
MDM2
Broad
Sanger
Nutlin-3a (-) (ln IC50)
8 6 4 2 0
R=0.5, p=9.0e-23
−2 −1 0
Scaled log2 FC
BAX
R=-0.38, p=3.3e-13
4 6 8
RNA-seq voom

Figure EV5.
